# Artificial Intelligence for Cardiac Diseases Diagnosis and Prediction Using ECG Images on Embedded Systems

**DOI:** 10.3390/biomedicines10082013

**Published:** 2022-08-19

**Authors:** Lotfi Mhamdi, Oussama Dammak, François Cottin, Imed Ben Dhaou

**Affiliations:** 1 Biotechnology Institute of Monastir, Environment Street, Monastir 5000, Tunisia; 2 Department of Mathematics, College of First Common Year, Um Al Qura University, Mecca 21955, Saudi Arabia; 3 CIAMS EA 4532, Paris-Saclay University, 91405 Orsay, France; 4 CIAMS EA 4532, Orleans University, 45067 Orleans, France; 5 Department of Computer Science, Hekma School of Engineering, Computing and Informatics, Dar Al-Hekma University, Jeddah 22246, Saudi Arabia; 6 Department of Technology, Higher Institute of Computer Sciences and Mathematics, University of Monastir, Monastir 5000, Tunisia

**Keywords:** ECG images, cardiac arrhythmia classification, healthcare, deep learning, Raspberry

## Abstract

The electrocardiogram (ECG) provides essential information about various human cardiac conditions. Several studies have investigated this topic in order to detect cardiac abnormalities for prevention purposes. Nowadays, there is an expansion of new smart signal processing methods, such as machine learning and its sub-branches, such as deep learning. These popular techniques help analyze and classify the ECG signal in an efficient way. Our study aims to develop algorithmic models to analyze ECG tracings to predict cardiovascular diseases. The direct impact of this work is to save lives and improve medical care with less expense. As health care and health insurance costs increase in the world, the direct impact of this work is saving lives and improving medical care. We conducted numerous experiments to optimize deep-learning parameters. We found the same validation accuracy value of about 0.95 for both MobileNetV2 and VGG16 algorithms. After implementation on Raspberry Pi, our results showed a small decrease in accuracy (0.94 and 0.90 for MobileNetV2 and VGG16 algorithms, respectively). Therefore, the main purpose of the present research work is to improve, in an easy and cheaper way, real-time monitoring using smart mobile tools (mobile phones, smart watches, connected T-shirts, etc.).

## 1. Introduction

In medicine and health care in general, electrophysiological signals provide information on the state of health. Among the electrophysiological data available, the electrocardiogram (ECG) turns out to be one of the most relevant and, therefore, worth studying. The variability of its cyclical behavior can provide evidence of emotional and behavioral disturbances and changes or cardiovascular pathologies. By studying their characteristics, these signals also remain relevant and non-invasive and help to provide effective diagnostics for heart disease.

Nowadays, there is a variety of portable gadgets which offer the possibility of detecting rare cardiac episodes due to their practical ambulatory properties, while ensuring continuous monitoring of the ECG during long periods. Detecting these episodes early in our daily lives increases the chance of a better lifestyle and comfort for humans.

Automatic detection in using signal processing and pattern recognition methods can constitute, therefore, an asset to provide relevant information without having recourse to hospital resources which are costly, time-consuming, and require much effort [1,2]. Also, with a better understanding of certain pathologies and the development of diagnostic methods and therapies allied to the evolution of technology in the medical field, better healthcare expectations have emerged in terms of efficiency. It is also clear that fast and personalized support is seen as the best reference. As a result, the demand for more efficient medical systems is increasing day by day.

In response, artificial intelligence (AI) has been used in fusion with the medical field by taking advantage of its ability to learn from a dense and complex database to find probable links between the various co-existing parameters. Those technologies are useful in assisting practicing physicians with the decision-making processes, not only in diagnosis but also in treatment, by monitoring patients and studying drug efficacy tests [3,4,5].

Heart abnormalities can be identified by the diagnosis of cardiac rhythm irregularities which are the main cause of cardiac arrhythmia. These abnormalities are responsible for anatomical changes in the atria and ventricles structure. Therefore, they produce changes in their activation, depolarization, and repolarization and ECG waveform morphology will change, causing irregularity [6].

In this same context, traditional methods of machine learning have become less reliable in detecting patterns and, therefore, in discerning pathological events. In order to tackle this problem, deep learning has been introduced in the medical field and has established itself with unparalleled efficiency [7,8]. ECG signals offer the possibility of using the Deep Neural Network (DNN) for pattern recognition and decision-making due to its cyclical behavior [9,10]. Thus, the genesis of artificial intelligence (AI) has opened new doors in several fields of science. This new way of reflecting human–machine interaction has proved to be ambitious and controversial and interest in it gradually increases each year with the evolution of automatic calculation technology [11].

It is important to note that the alarming situation of deaths caused by cardiovascular diseases (CVD), or sudden deaths, remain very worrying on a global scale. This pushes scientific research to find a better way to anticipate these direct consequences. In this context, early detection of arrhythmia is an important clinical step that can save lives. The commonest method to detect cardiac arrhythmia uses the electrocardiogram (ECG), which measures the electrical activity of the heart.

The main objective of the present work is to develop an automated technique for the diagnosis of cardiac arrhythmia and, thereafter, to implement it on an embedded system. We performed a two-category classification of ECG recordings (cardiac arrhythmia, and healthy person) by using ECG images from the publicly available arrhythmia database published by Khan’s team [12,13].

Our study will explore the technique of deep learning using deep convolutional neural network (CNN) models, first to detect cardiovascular disease from images of electrocardiogram (ECG) tracings and, second, to predict any type of cardiac arrhythmia. For this purpose, we used trained algorithms and, thereafter, we implemented the most suitable one, subsequently, on a Raspberry Pi 4B 8 Go. We worked with deep-learning pretrained models (MobileNet V2 and VGG16) using CNN with Tensorflow and Keras as backend.

Therefore, the objective of this work is as follows:

- construction of a deep-learning algorithm model for application to ECG signals;
- detection and classification of abnormal patterns (spectra) on ECG signals; and
- validation and improvement of the predictive model on a new database.

## 2. Materials and Methods

Our goal was to develop an algorithm able to detect and classify data into two different categories (normal for healthy people and cardiac arrhythmia for persons with cardiac pathologies). The learning will be directly on the ECG data without any significant preprocessing.

### 2.1. Data Used

In this study we used a newly released public database [12,13]. It has about 928 images of ECG tracings, representing four different subject classes belonging to cardiac arrhythmia category:

- Normal: for subjects who do not suffer from any pathology;
- MI: for subjects with a myocardial infarction (MI);
- HMI: for subjects with a history of myocardial infarction (recovered from a MI); and
- ABH: for subjects suffering from cardiac arrhythmias (abnormal heartbeat).

Ultimately, we will have 741 ECG images for the training data and 187 for the test data.

For this purpose, we conducted two pre-trained deep neural networks (DNNs), MobilNetV22 and VGG16, which have been adapted with our data for better performance. Various parameters have been changed (number and type of layers, number of filters per layer, dropout, batch size, learning rate, etc.) to find the best desired model. The database was divided into training and testing data, with a rate of 80% and 20%, respectively. The breakdown of the total number of ECG images used for each class is shown in Table 1.

### 2.2. Preprocessing

Before passing the images to the model for training, we resized them (244.244), then normalized (1/255) to adapt them to the requirements of the algorithm. It is very important to note that it has been shown that deep-learning technique requires significantly more training data than other machine-learning approaches [14]. In case the images and few data are available, the augmentation technique can be used to duplicate images to increase the amount of data. Indeed, this technique makes it possible to generate new versions of the same image by applying different image-processing operations (zoom, stretch, contrast, etc.). However, it is not always useful and can distort learning if you change some parameters necessary for image recognition.

In this paper, we have made the choice of six image processing parameters (brightness, contrast, gamma, hue, saturation, and central-crop) without affecting the information and avoiding distorting the learning of the algorithm. The new images resulting from the augmentation technique will be taken into account by the model at the time of training; this is an online augmentation that does not require saving the new data to the computer hard drive. We compared various image-processing parameters, the best ones with better performance made to images during augmentation are as follows:

- brightness (0.2): brightness adjustment;
- contrast (0.6): contrast adjustment;
- gamma (gamma = 3, gain = 2): control of the overall brightness and the blue-green–red of the image;
- hue (0.9): hue that controls the colors red, yellow, green, and blue;
- saturation (0.2): adjustment of the hue–white light mixture; and
- central-crop (0.92): division of the area of interest.

### 2.3. Hyper-Parameters Used

Hyper-parameters are pre-defined parameters provided to the algorithm to control learning and increase its performance. We can cite the learning rate, the number and size of the hidden layers, etc. In the present study, we have fixed the value of some hyper-parameters as follows:

- learning rate (0.01);
- dropout (0.1);
- Conv2D filters (min = 64, max = 128, step = 32);
- batch size = 32; and
- optimizer (Adam(1 × 10^−5^)).

We chose moderately sized architectures for both DNN models. For each experiment, less than 100 epochs were enough to reach an excellent accuracy. We ran several trials of experiment with a CNN and pooling layer, including number of filters 32, 64, and 128. We used 0.01 as the best efficient learning rate for all deep-learning architectures for our experiments, after considering other learning-rate tests and the factors of training time and computational cost.

**Figure 1:**
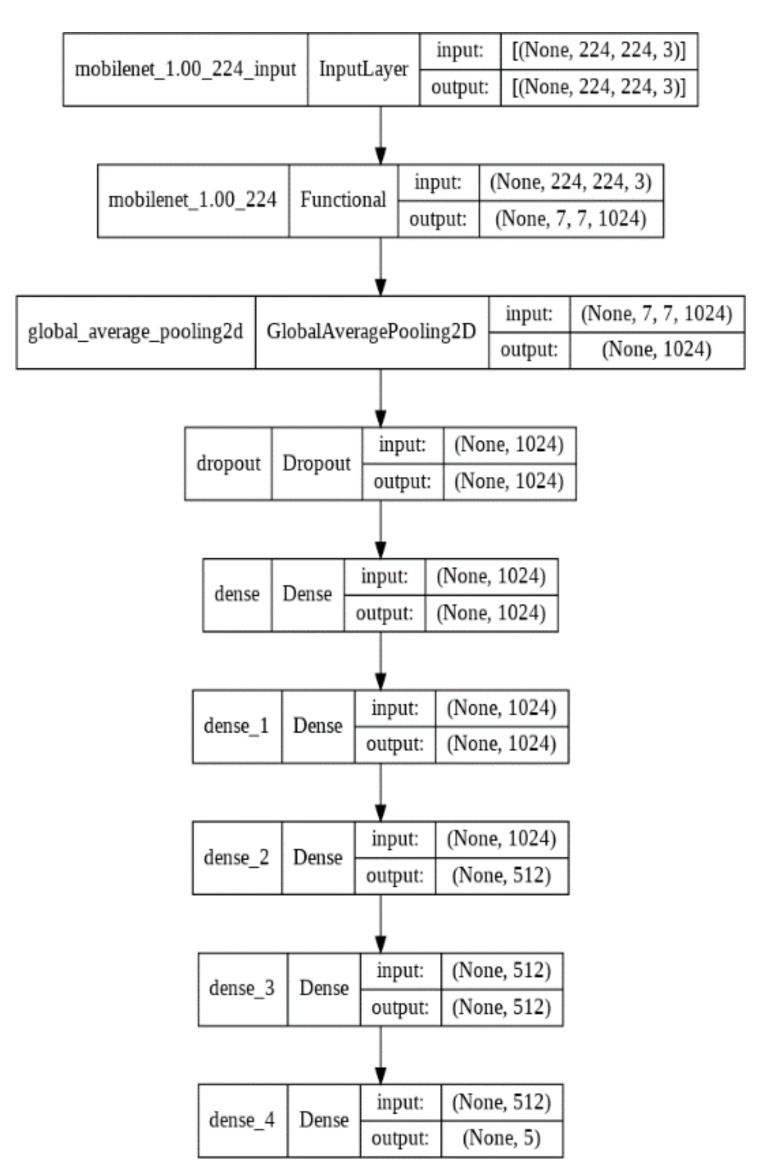
MobileNet V2 architecture for transfer learning.

**Figure 2:**
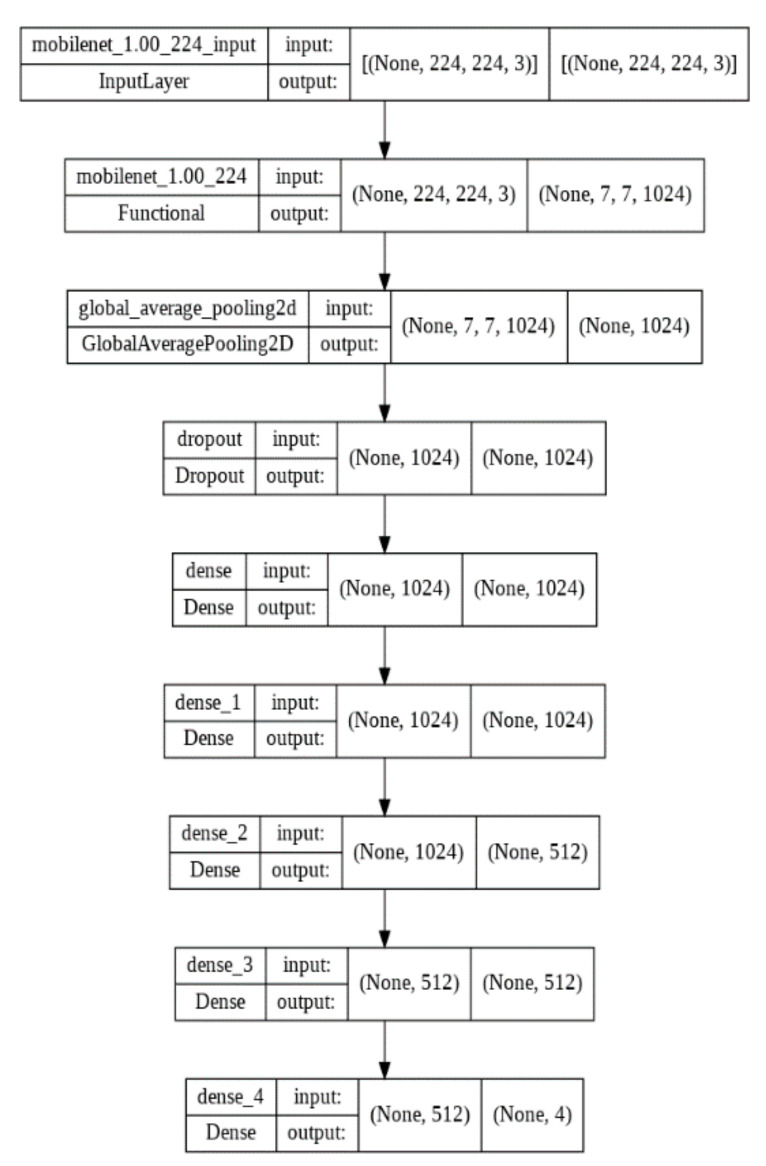
MobileNet V2 architecture for fine-tuning.

**Figure 3:**
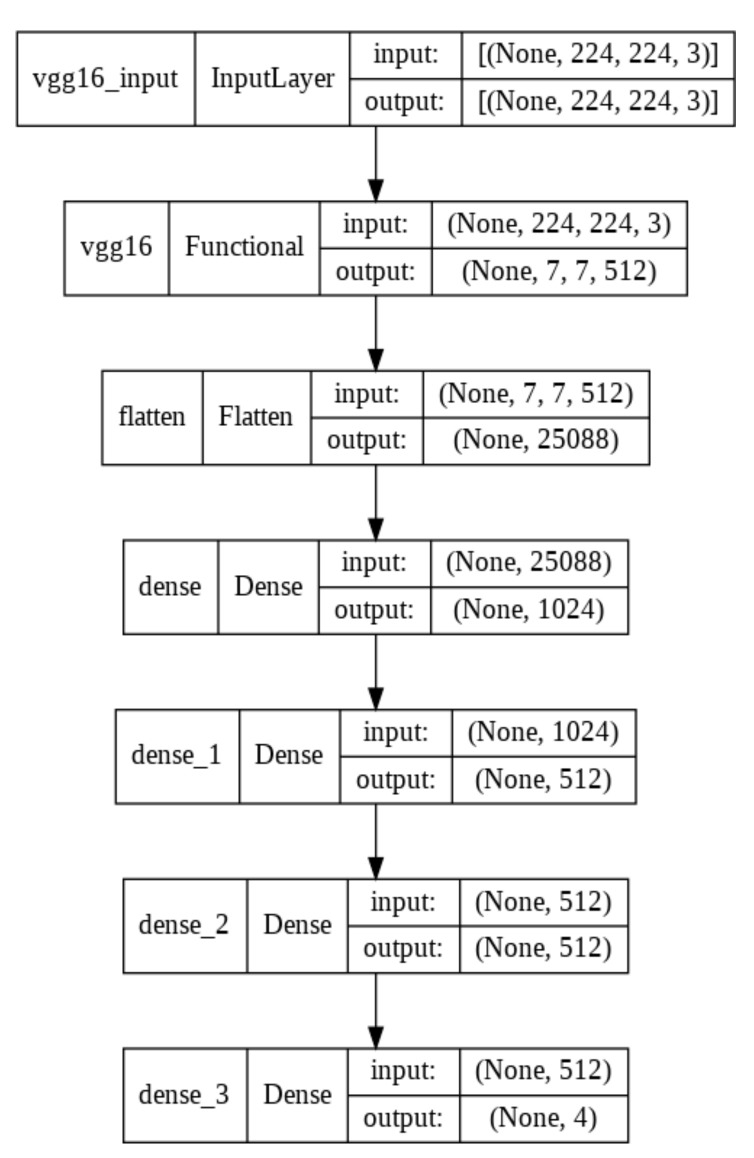
VGG16 architecture for transfer learning.

**Figure 4:**
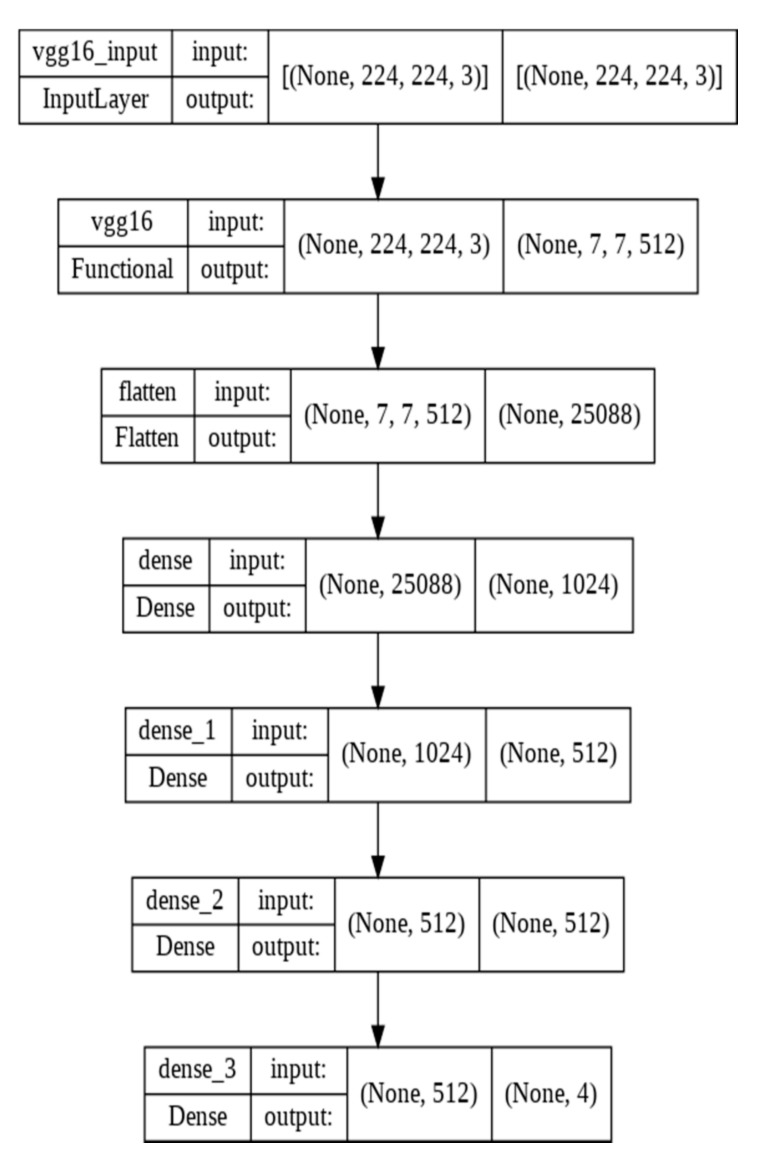
VGG16 architecture for fine-tuning.

We tried different network architectures to find the optimum one for the input data we used, and we chose two CNN models which are the most suitable for our study. The corresponding architectures for both models are shown in Figure 1, Figure 2, Figure 3 and Figure 4.

### 2.4. Network Architecture

In this study, we used a single classification scheme with four classes (normal, myocardial infarction, recovered myocardial infarction, and abnormal heart rhythm).

We performed two deep-learning algorithms (MobileNet V2 and VGG16) to classify ECG images and predict cardiac arrhythmia. The corresponding MobileNet V2 model architecture representations are shown in Figure 1 and Figure 2 for transfer learning and fine-tuning steps, respectively. VGG16 architecture representations are shown in Figure 3 and Figure 4. They correspond, respectively, to transfer learning and fine-tuning step.

We split the data into 80% for training and 20% for tests. Our algorithms will serve as basic models for classification and prediction. For each model studied, the following steps were followed:

- We applied transfer learning by taking the classic pre-trained model chosen without its last classification layer (Table 2). We replaced this layer by another one, more adapted to the new data; and
- We applied fine-tuning process and we made some adjustments to increase the model performance after the transfer learning step. The idea was to retrain the previously used transfer learning model after making some modification on its layers numbers, types, and new hyper parameters (learning rate, batch size, activation, and optimizer). In our case, we excluded the six last layers from any adjustment and modified only the rest of our model by applying the learning process only on the last 23 layers (Table 3).

Regarding the VGG16 model, the adjustment was applied to the entire transfer learning model (Table 4), excluding the last six layers, too. Then we trained only the last nine layers to perform the fine-tuning step (Table 5).

We trained both DNN to detect normal cardiac rhythm as well as abnormalities and to make predictions, thereafter. The model’s parameters were learned using only the training set and the design choices depended on maximizing the performance on the validation data set. We used Keras and TensorFlow as backend, dedicated for deep-learning processing and implemented in Python, and we ran the training data and test data on “Google Colab” cloud service.

Deep-learning algorithms contain different parameters, such as learning rate and number of units, which heavily affect performance [15]. Indeed, it is very important to choose optimal values for these parameters. In this work, we tried several trails of experiments to identify the best ones.

In our study, the learning process went very well and there was no need to use the automating hyper-parameters technique—Keras tuner, which takes too long.

### 2.5. Performance Metrics

In this paper, two deep-learning CNN models were developed and evaluated using the classification report, which gives us accuracy values, recall, and the f1-score. Accuracy is the measure of the total number of correct predictions among all predictions. Recall shows false-negative occurrence; it is a sign of the failure of the model to predict. In other words, it is the correct prediction of actual positive cases. F1-score is an average of recall and precision.

Our method performance was evaluated with five metrics (sensitivity, specificity, accuracy, precision, and f1-score) given in equations below [16]:

$$\mathrm{Sensitivity}=\frac{TP}{\left(TP+FN\right)}$$

$$\mathrm{Specificity}=\frac{TN}{\left(TN+FP\right)}$$

$$\mathrm{Accuracy}=\frac{\left(TP+TN\right)}{\left(TP+FP+FN+TN\right)}$$

$$\mathrm{Precision}=\frac{TP}{\left(TP+FP\right)}$$

$$F1-\mathrm{score}=\frac{2*\left(Sensitivity*Precision\right)}{\left(Sensitivity+Precision\right)}$$

where *TP*, *TN*, *FP,* and *FN* are, respectively, true positive, true negative, false positive, and false negative detected ECG images. The confusion matrix, corresponding to each learning, was also drawn up to give us an idea about the sensitivity and specificity of the algorithm when predicting the test data.

After learning, the model with the best accuracy will be converted to a portable version (TensorFlow Lite) and implemented on a Raspberry Pi 4B 8GB.

### 2.6. Raspberry Pi

The implementation of the studied models was made for the purpose of a later use on connected objects. We used a Raspberry Pi 4B 8Go, given its robustness which allows it to work with algorithms of this type. It is an ARM-based single-board nano-computer.

## 3. Results

We used MobileNet V2 and VGG16 algorithms for cardiovascular-disease diagnosis and prediction. We calculated the accuracy corresponding to each of the four classes, to evaluate and compare both models. Our work showed excellent accuracy; this is very important for easily detecting and differentiating the different classes. The highest accuracy was found in the case of Myocardial Infarction (MI) and Previous History of MI (HMI) for both models. A summary of the performance metrics found for every class, in both models, is given in Section 3.2.

### 3.1. Data Augmentation

**Figure 5:**
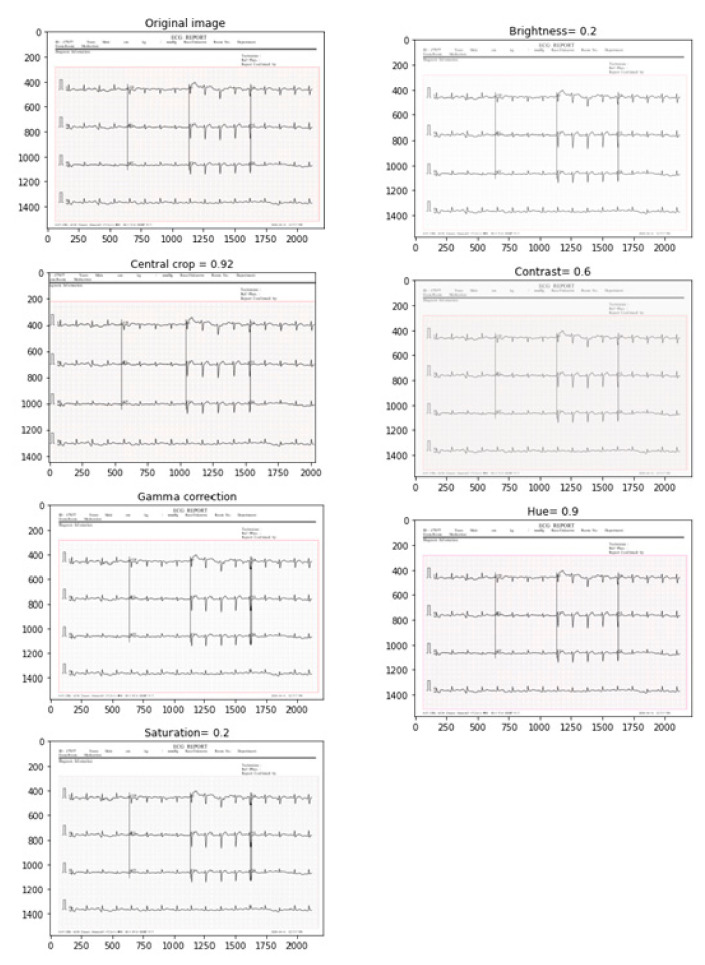
ECG images modifications after the data augmentation process.

The data augmentation result is given on the image plate below (Figure 5). Images are easily readable without affecting their information. The learning process can be performed on the go with the newly-built ECG images without any fear of missing any information needed for better learning.

### 3.2. Classification Report for MobileNet V2 and VGG16

To evaluate the model, performance metrics values for each class are given in the tables below (Table 6 and Table 7) for MobileNet V2.

Accuracy has increased from 0.93 to 0.95 after the fine-tuning step. It represents a small evolution but is very useful for getting better performance from the model.

Concerning the VGG16 model, classification reports are shown in Table 8 and Table 9. One can see the same accuracy value for both models after the fine-tuning step (0.95). On the other hand, the accuracy increased better when we applied the fine-tuning process on the VGG16 model (from 0.91 to 0.95) compared to the one with MobileNet V2 (from 0.93 to 0.95).

### 3.3. Confusion Matrix

The confusion matrix is a representation of the five metrics (true positive, false positive, false negative, and true negative) used to evaluate the model performance in every performed class.

**Figure 6:**
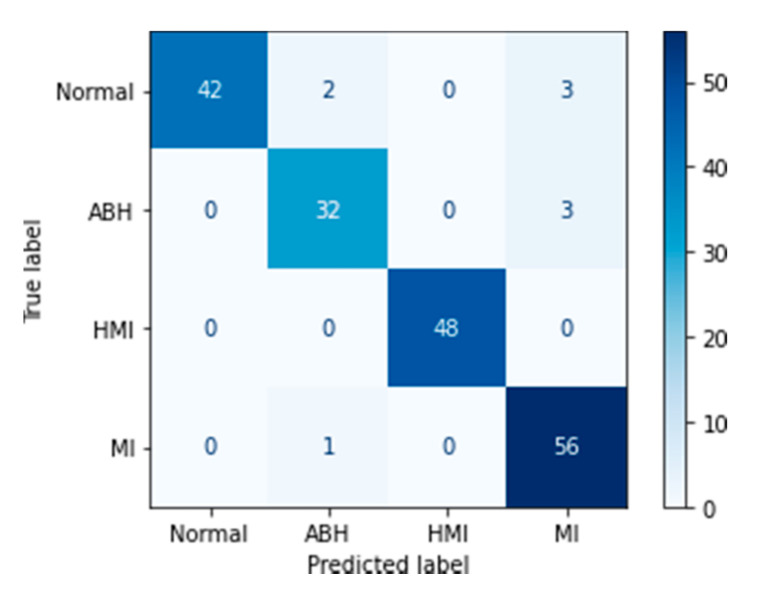
Confusion matrix for the MobileNet V2 model.

In the present work, looking on the confusion matrix corresponding to MobileNet V2 after the fine-tuning step (Figure 6), the myocardial infarction (MI) class showed the highest average score of about 56. On the other hand, the abnormal heartbeat class (ABH) presented the smallest average score of about 32.

**Figure 7:**
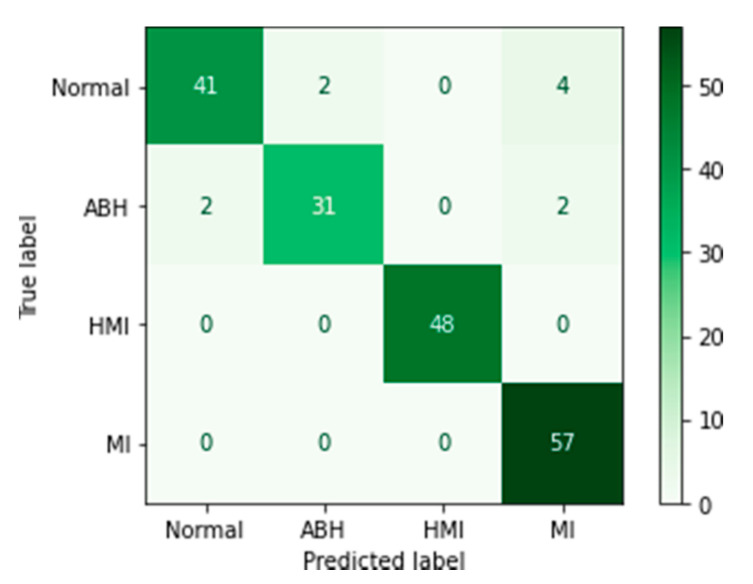
Confusion matrix for the VGG16 model.

Concerning VGG16 after the fine-tuning step, we found the same observations as for the MobileNet V2 model (myocardial infarction class with the highest average score of about 57 and abnormal heartbeat class (ABH) with the smallest average score of about 31) (Figure 7).

In order to demonstrate the reproducibility of the model architecture to new data, we tested it on the test data. The model performance demonstrated excellent results and, therefore, one can make conclusions about the ability of our DNN-based approach to generalize to a new set of ECG records from a different data.

### 3.4. Raspberry Pi

We evaluated the performance of MobileNet V2 and VGG16 using different evaluation metrics. The accuracy reached 95% for both models. After implementing the TFLite version (portable version), for both models, on a Raspberry Pi 4 B, 8Go, the prediction on the test data showed excellent accuracy values equal to 94% and 92%, with an execution time of 0.16 and 0.24 s for MobileNet V2 and VGG16, respectively. Moreover, an optimized version of both TFLite models will be studied in our next work. Indeed, this version will have a smaller size to gain faster speed on wearable devices without losing performance.

## 4. Discussion

In recent papers, the state-of-the-art study on deep learning has shown satisfactory results in detection, classification, and prediction tasks on medical images in general, and on ECG in particular. Indeed, cardiac arrhythmia is the result of anomalies occurring in the heart. These anomalies are reflected as deviation of ECG waveform from its normal shape and size and cause abnormal activation, depolarization, and repolarization, after anatomical changes in the structure of atria and ventricles.

In a recent study [17], the authors used 10,000 recorded ECG images from a public arrhythmia database and performed two different class scenarios. They used a one-dimensional convolutional neural network (1D-CNN) and a long short-term memory network (LSTM) block was added to this model for sequence learning. After training, the accuracy values achieved for the reduced and merged rhythm classes were about 92.24% and 96.13%, respectively. This combination gave better performances in other different works [18,19,20].

In another study [21], the authors used the MIT-BIH arrhythmia database. They obtained five-fold cross-validation accuracy of 0.834 in distinguishing normal and abnormal cardiac arrhythmia, from ECG images with CNN-LSTM. Moreover, according to these authors, the accuracy obtained by other hybrid architectures of deep-learning algorithms was comparable to the CNN-LSTM.

Safdarian et al. performed a study about the myocardial infarction (heart attack), a dangerous cardiac pathology [22]. They used a data from a single lead ECG myocardial (MI) and achieved about 94.74% accuracy. Sharma et al. conducted a multiscale eigenspace analysis on 12-lead ECG data and the accuracy was about 96% [23].

Mohammedzadeh et al. achieved an excellent accuracy of 99.38% with an MIT BIH data set for arrhythmia classification [24]. In another study, the ECG arrhythmia MIT database was classified using artificial neural network (ANN) with an accuracy of about 96.77% [25].

In a recent study [26], the authors presented a DNN model trained in a data set with more than two million labeled exams, for recognizing six types of abnormalities in 12-lead ECG recordings, and representative of both rhythmic and morphological ECG abnormalities. They obtained f1-scores above 80% and specificity over 99%. Compared to analysis studied in a single-lead setup, the present results show ECG analysis based on DNNs generalizes well to 12-lead exams, giving an excellent result very closer to the standard clinical practice. Moreover, Hannun et al. developed a deep neural network (DNN) to classify 12 cardiac rhythm classes using about 91,000 single-lead ECGs [27]. The results found showed a ROC (Receiver Operating characteristic Curve) of 0.97 and an average f1-score of 0.83. They demonstrated that the deep-learning model used was able to classify different arrhythmia types from single-lead ECGs, with high performance closer to that of cardiologists.

Baloglu et al. developed an efficient automated deep-learning model to distinguish between 10 different myocardial infarction classes and normal ECG records on 12-lead ECG signals [28]. They used an open-access Physiobank (PTB) ECG database and found a classification performance over 99%. This model is easily implementable on portable healthcare devices.

Acharya et al. proposed a method able to detect cardiac abnormalities and classify them to four different classes [29]. The classification results achieved were about 97.98%, 99.61%, and 94.84% for accuracy, sensitivity, and specificity, respectively.

In a recent paper, the authors proposed a data augmentation method called RandECG to classify ECG with deep neural networks (DNN) [30]. They applied various transformation techniques and selected the suitable ones for ECG. Tested on two different data sets, the efficacy of RandECG has been improved up to 3.51% compared to the data sets before augmentation.

Usually, ECGs are recorded on paper, which can be noisy. Therefore, digitizing these records into a high-quality readable signal is critical for diagnosis and further analysis [31]. In this paper, the authors proposed a powerful deep-learning approach for ECG digitization. The approach achieved excellent segmentation and very high concordance with the first true signal.

Du et al. suggested a fine-grained multi-label ECG framework (FM-ECG) able to find out the critical fine-grained parts from the ECG records and further abnormalities [32]. This framework has shown an excellent performance of making multi-label classification in ECG images. The authors used CNN-RNN (Recurrent Neural Network)-like framework, which utilizes CNNs to extract important parts via fine-grained learning, and uses RNNs to recurrently infer the abnormal categories considering the class dependencies. They used 12-lead ECG images from two different data sets. The experimental results demonstrated that FM-ECG is an excellent framework to help clinicians to detect abnormalities from ECG records.

The project of Koshti et al. consists of a monitoring system for detecting cardiac abnormalities from the MIT-BIH database [33]. The system analyses the signal, extracts features, and detects abnormal conditions (arrhythmia). Thereafter, Raspberry Pi 4B sends the results of the ECG signals to the web server. The framework used achieved about 95.4% success rate.

In Cheikhrouhou’s team’s work [34], the authors proposed a modular 1D-CNN approach, which uses a trained machine-learning model for predicting cardiac abnormalities, based on ECG records captured from IoT (Internet of Things) wearable devices. This approach achieved 99.46% of accuracy using the MIT-BIH Arrhythmia database. They applied the GridSearch algorithm with the cross-validation method.

Patil and Bhole presented a real-time ECG monitoring system using the Raspberry Pi system [35]. Indeed, ECG records can be viewed at any place at any time. This important concept is used to detect the heart defect and abnormalities in ECG images, without involving an expensive ECG machine nor automatic human intervention. The availability of real-time patient’s data anywhere anytime helps to take a quick diagnosis and, therefore, to make more efficient and faster decisions which are crucial to saving patients’ lives.

Granados et al. proposed an IoT (Internet of Things) platform; useful to implement a real-time analysis and the use of cloud deep neural network, for cardiovascular diagnosis through ECG classification [36]. The major goal of this study is to use 12-lead ECG signals to diagnose cardiovascular diseases, as well as different types of correlated abnormalities. ECG classification was performed by a CNN classifier and Tensorflow library, implemented on a GPU-accelerated machine. This IoT platform is very useful to increase healthcare services efficiency, by conducting home treatment instead of using valuable hospital resources. Therefore, these results can be used to ensure better lifestyle habits for people.

It is well known that preprocessing steps are of great importance in reaching an excellent image classification. Abadi’s team assumes that deep-learning techniques require significantly more training data [14]. In the case of few images being available, the augmentation technique can be used to duplicate images to increase the amount of data. Therefore, data augmentation is used for improving classification performance, especially in images. It should increase data amount and diversity by adding random changes based on augmentation techniques. In this context, Nonaka and Seita explored a data augmentation technique suitable for ECG data [30]. In fact, this technique has successfully improved atrial fibrillation classification with single-lead ECG data, with no need to change the DNN architecture. The authors used the public CinC/Challenge 2017 data set, which consists of 8528 samples classed to four classes. The results found shown that the ECG augmentation is effective in classifying atrial fibrillations. Moreover, the classification accuracy has been improved.

Compared to all these studies, our present work has shown remarkable results on the detection, classification, and prediction of the four classes studied and achieved high accuracy. Furthermore, model evaluation with the test dataset has achieved results comparable to those made by clinicians. Therefore, the performed models could be used to help clinicians for cardiac diagnosis and decision-making. However, we should note that none of our performed deep-learning CNN algorithms may substitute for clinicians’ handiwork.

In addition, there are several limitations for this study. Indeed, we used a small database for training and testing steps, which is insufficient regarding the thousands of hyper-parameters in the deep-learning approach. Moreover, an independent test dataset is missing which is considered another limitation. Furthermore, when selecting deep-learning hyper-parameters, we did not take into account optimization techniques.

## 5. Conclusions

Cardiac abnormalities may cause dangerous damages to the heart and even lead to death. Therefore, their rapid and accurate diagnosis is important to avoid deaths. For this purpose, clinicians had recourse to ECG interpretation which is critical for diagnosing cardiac arrhythmia and requires expertise and is time-consuming. Thus, automatic diagnosis using computers may be useful for this task.

In this paper, we proposed a new automatic deep-learning model to diagnose, classify, and predict cardiac arrhythmias based on 12-lead ECG images interpretation. We used the convolutional neural network (CNN), a very famous deep-learning technique for images classification. Importantly, both trained models with the proposed architecture achieved excellent performance with an accuracy over 95.00%. Therefore, with this performance, we envisage using these models for automated diagnosis in intensive-care units and wearable devices for better health-care monitoring.

In future studies, we will evaluate the model performance on the different cardiac arrhythmia’s datasets. Moreover, we will use the ECG signals of various leads to find the exact affected heart location. Further work will consider using more deep-learning techniques and hyper-parameter optimization approaches.

## Figures and Tables

**Table 1:** Data description.

Class	Training Dataset	Test Dataset	Total Dataset
Normal	227	57	284
MI	191	48	239
HMI	137	35	102
ABH	186	47	233
Total dataset	741	187	928

**Table 2:** MobileNet Transfer Learning summary.

Layer Type	Output Shape	Param
mobilenet_1.00_224 (Functional)	(None, 7, 7, 1024)	3,228,864
global_average_pooling2d	(None, 1024)	0
dropout (Dropout)	(None, 1024)	0
dense (Dense)	(None, 1024)	1,049,600
dense_1 (Dense)	(None, 1024)	1,049,600
dense_2 (Dense)	(None, 512)	524,800
dense_3 (Dense)	(None, 512)	262,656
dense_4 (Dense) Total params: 6,118,085 Trainable params: 2,889,221	(None, 5)	2565
Non-trainable params: 3,228,864		

**Table 3:** MobileNet Fine Tuning summary.

Layer Type	Output Shape	Param
mobilenet_1.00_224 (Functional)	(None, 7, 7, 1024)	3,228,864
global_average_pooling2d	(None, 1024)	0
dropout_1 (Dropout)	(None, 1024)	0
dense_5 (Dense)	(None, 1024)	1,049,600
dense_6 (Dense)	(None, 1024)	1,049,600
dense_7 (Dense)	(None, 512)	524,800
dense_8 (Dense)	(None, 512)	262,656
dense_9 (Dense) Total params: 6,117,572 Trainable params: 2,888,708	(None, 4)	2052
Non-trainable params: 3,228,864		

**Table 4:** VGG16 Transfer Learning summary.

Layer Type	Output Shape	Param
vgg16 (Functional)	(None, 7, 7, 512)	14,714,688
flatten_1 (Flatten)	(None, 25,088)	0
dense_4 (Dense)	(None, 1024)	25,691,136
dense_5 (Dense)	(None, 512)	524,800
dense_6 (Dense)	(None, 512)	262,656
dense_7 (Dense) Total params: 41,195,332 Trainable params: 26,480,644, non-trainable params: 14,714,688	(None, 4)	2052

**Table 5:** VGG16 Fine Tuning summary.

Layer Type	Output Shape	Param
vgg16 (Functional)	(None, 7, 7, 512)	14,714,688
flatten_1 (Flatten)	(None, 25,088)	0
dense (Dense)	(None, 1024)	25,691,136
dense_1 (Dense)	(None, 512)	524,800
dense_2 (Dense)	(None, 512)	262,656
dense_3 (Dense) Total params: 41,195,332 Trainable params: 26,480,644, non-trainable params: 14,714,688	(None, 4)	2052

**Table 6:** Classification report for MobileNet V2 transfer learning.

	Precision	Recall	f1-Score	Support
Normal	0.95	0.87	0.91	47
Abnormal heartbeat (ABH)	0.88	0.86	0.87	35
Previous history of MI (HMI)	0.98	1.00	0.99	48
Myocardial infarction (MI)	0.90	0.96	0.93	57
accuracy			0.93	187
macro avg	0.93	0.92	0.93	187
weighted avg	0.93	0.93	0.93	187

**Table 7:** Classification report for MobileNet V2 fine-tuning.

	Precision	Recall	f1-score	Support
Normal	1.00	0.89	0.94	47
Abnormal heartbeat (ABH)	0.91	0.91	0.91	35
Previous history of MI (HMI)	1.00	1.00	1.00	48
Myocardial infarction (MI)	0.90	0.98	0.94	57
accuracy			0.95	187
macro avg	0.95	0.95	0.95	187
weighted avg	0.95	0.95	0.95	187

**Table 8:** Classification report for VGG16 transfer learning.

	Precision	Recall	f1-score	Support
Normal	0.95	0.77	0.85	47
Abnormal heartbeat (ABH)	0.91	0.89	0.90	35
Previous history of MI (HMI)	0.89	1.00	0.94	48
Myocardial infarction (MI)	0.90	0.96	0.93	57
accuracy			0.91	187
macro avg	0.91	0.90	0.90	187
weighted avg	0.91	0.91	0.91	187

**Table 9:** Classification Rerort for VGG16 fine-tuning.

	Precision	Recall	f1-score	Support
Normal	0.95	0.87	0.91	47
Abnormal heartbeat (ABH)	0.94	0.89	0.91	35
Previous history of MI (HMI)	1.00	1.00	1.00	48
Myocardial infarction (MI)	0.90	1.00	0.95	57
accuracy			0.95	187
macro avg	0.95	0.94	0.94	187
weighted avg	0.95	0.95	0.95	187

## Data Availability

The database used is free and published for public use on the following links: https://data.mendeley.com/datasets/gwbz3fsgp8/1; https://data.mendeley.com/datasets/gwbz3fsgp8/2 (accessed on 15 November 2021).

## Abbreviations

The following abbreviations are used in this manuscript:

ECG	Electrocardiogram
AI	Artificial Intelligence
DL	Deep Learning
CVD	Cardiovascular Diseases
CNN	Convolutional Neural Network
MI	Myocardial infarction
HMI	History of Myocardial Infarction
ABH	Abnormal Heartbeat
DNN	Deep Neural Network
TP	True Positive
TN	True Negative
FP	False Positive
FN	False Negative
LSTM	Long Short-Term Memory Network
RNN	Recurrent Neural Network
IoT	Internet of Things
